# ^18^F-labeled magnetic nanoparticles for monitoring anti-angiogenic therapeutic effects in breast cancer xenografts

**DOI:** 10.1186/s12951-019-0534-7

**Published:** 2019-10-11

**Authors:** Yanshu Wang, Huanhuan Liu, Defan Yao, Jinning Li, Shuyan Yang, Caiyuan Zhang, Weibo Chen, Dengbin Wang

**Affiliations:** 1 Department of Radiology, Xinhua Hospital, Shanghai Jiao Tong University School of Medicine, Shanghai, 200092 China; 2 Philips Healthcare, Shanghai, 200233 China

**Keywords:** PET/MRI, Dual modality, α_v_β_3_-integrin, Anti-angiogenesis therapy, Breast cancer

## Abstract

**Purpose:**

To develop a novel fluorine-18 (^18^F)-labeled arginine–glycine–aspartic acid (RGD)-coupled ultra-small iron oxide nanoparticle (USPIO) (hereafter, referred to as ^18^F-RGD@USPIO) and conduct an in-depth investigation to monitor the anti-angiogenic therapeutic effects by using a novel dual-modality PET/MRI probe.

**Methods:**

The RGD peptide and ^18^F were coupled onto USPIO by click chemistry. In vitro experiments including determination of stability, cytotoxicity, cell binding of the obtained ^18^F-RGD@USPIO were carried out, and the targeting kinetics and bio-distribution were tested on an MDA-MB-231 tumor model. A total of 20 (n = 10 per group) MDA-MB-231 xenograft-bearing mice were treated with bevacizumab or placebo (intraperitoneal injections of bevacizumab or a volume-equivalent placebo solution at the dose of 5 mg/kg for consecutive 7 days, respectively), and underwent PET/CT and MRI examinations with ^18^F-RGD@USPIO before and after treatment. Imaging findings were validated by histological analysis with regard to β_3_-integrin expression (CD61 expression), microvascular density (CD31 expression), and proliferation (Ki-67 expression).

**Results:**

Excellent stability, low toxicity, and good specificity to endothelial of ^18^F-RGD@USPIO were confirmed. The best time point for MRI scan was 6 h post-injection. No intergroup differences were observed in tumor volume development between baseline and day 7. However, ^18^F-RGD@USPIO binding was significantly reduced after bevacizumab treatment compared with placebo, both on MRI (P < 0.001) and PET/CT (P = 0.002). Significantly lower microvascular density, tumor cell proliferation, and integrin β_3_ expression were noted in the bevacizumab therapy group than the placebo group, which were consistent with the imaging results.

**Conclusion:**

PET/MRI with the dual-modality nanoprobe, ^18^F-RGD@USPIO, can be implemented as a noninvasive approach to monitor the therapeutic effects of anti-angiogenesis in breast cancer model in vivo.

## Introduction

Breast cancer is the leading cause of cancer-related deaths among women of all ages [1]. Angiogenesis plays a key role in the growth and metastasis in breast cancer, which has provided a strong rationale for using antiangiogenic therapies [2]. Bevacizumab is an anti-vascular endothelial growth factor (VEGF) monoclonal antibody, and has been one of the attractive angiogenesis inhibitors in preclinical and clinical trials. Increasing evidence has indicated that bevacizumab improves the efficacy of chemotherapy in invasive breast cancer, with data derived both from the metastatic and neoadjuvant settings [3]. Although bevacizumab has been found for clinical applications, its limited efficacy on the overall survival benefits and resistance pose unresolved challenges. A previous study even raised concerns that antiangiogenic therapy might fuel cancer invasiveness and metastasis by aggravating intratumoral hypoxia and creating a proinflammatory environment [4]. Accordingly, these studies are invaluable for assessing the early therapeutic effects of bevacizumab and identifying the subpopulation of patients who would most appropriately benefit from bevacizumab treatment, with concerns about avoiding side effects [5], drug resistance, and high costs. However, with regard to detecting tumor responses to bevacizumab within a short time period, histopathological techniques are typically invasive and do not provide valuable information about the function of tumor vessels, and the measurement of tumor size seems to lack reliability for bevacizumab’s cytostatic effect, given that it is relatively sensitive to chemotherapy-induced cytotoxicity [6].

Using target-specific probes, molecular imaging can visualize and quantify therapeutic effects of anti-angiogenic treatment by monitoring some of the molecular and signaling pathway changes involved in angiogenesis. Integrin a_v_β_3_, which is overexpressed on activated and proliferated endothelial cells and involved in neo-angiogenic signaling cascades including the VEGF pathway [7], has been represented as a potential molecular marker for angiogenesis. The RGD sequence can specifically and strongly bind to a_v_β_3_-integrin [8], and significant progress has been made by using RGD-based probes to detect suitability for early anti-angiogenic treatment with modalities, including PET [9–13], SPECT [13, 14], MRI [15, 16], and ultrasound imaging [17]. However, no single modality can allow obtaining all the required information for anti-angiogenic assessment.

Combination of PET and MRI molecular imaging modalities can offer synergistic advantages over each single modality alone for monitoring the early anti-angiogenic efficacy: in addition to the extremely high sensitivity and quantitation capability for a_v_β_3_-integrin expression provided by PET, MR imaging can correct the partial volume effect of PET and aid in highly accurate image registration. Moreover, RGD-based radiotracers are sensitive to metastasis [18, 19]; MRI with USPIO may enhance detection of the tumor vessel’s structural and size changes in antiangiogenic therapy [20, 21]. Imaging with PET/MRI probe during antiangiogenic therapy may reveal contributions of the vascular phenotypes to metastatic individual tumors, which might provide the basis for improving the applicability and reach of anti-angiogenic cancer therapies. Furthermore, multi-parametric MRI sequences can provide a range of functional information reflected by changes in perfusion and metabolism in anti-angiogenic treatment. The fusion of PET and MRI agents into a single probe can embrace all the above advantages by “single pharmacological behavior” for both imaging acquisitions [22] and avoid differential bio-distribution, which is more beneficial for temporal and spatial correlation of the two imaging modalities [23].

Thus, we aimed to synthesize and test a novel ^18^F-labeled RGD-coupled polyacrylic acid (PAA)-coated USPIO (referred to as ^18^F-RGD@USPIO) PET/MRI nanoprobe, for in vivo monitoring of anti-angiogenic efficacy in MDA-MB-231 breast cancer xenografts treated with bevacizumab over 1 week.

## Material and method

### Synthesis and characterization of ^18^F-RGD@USPIO

**Fig. 1:**
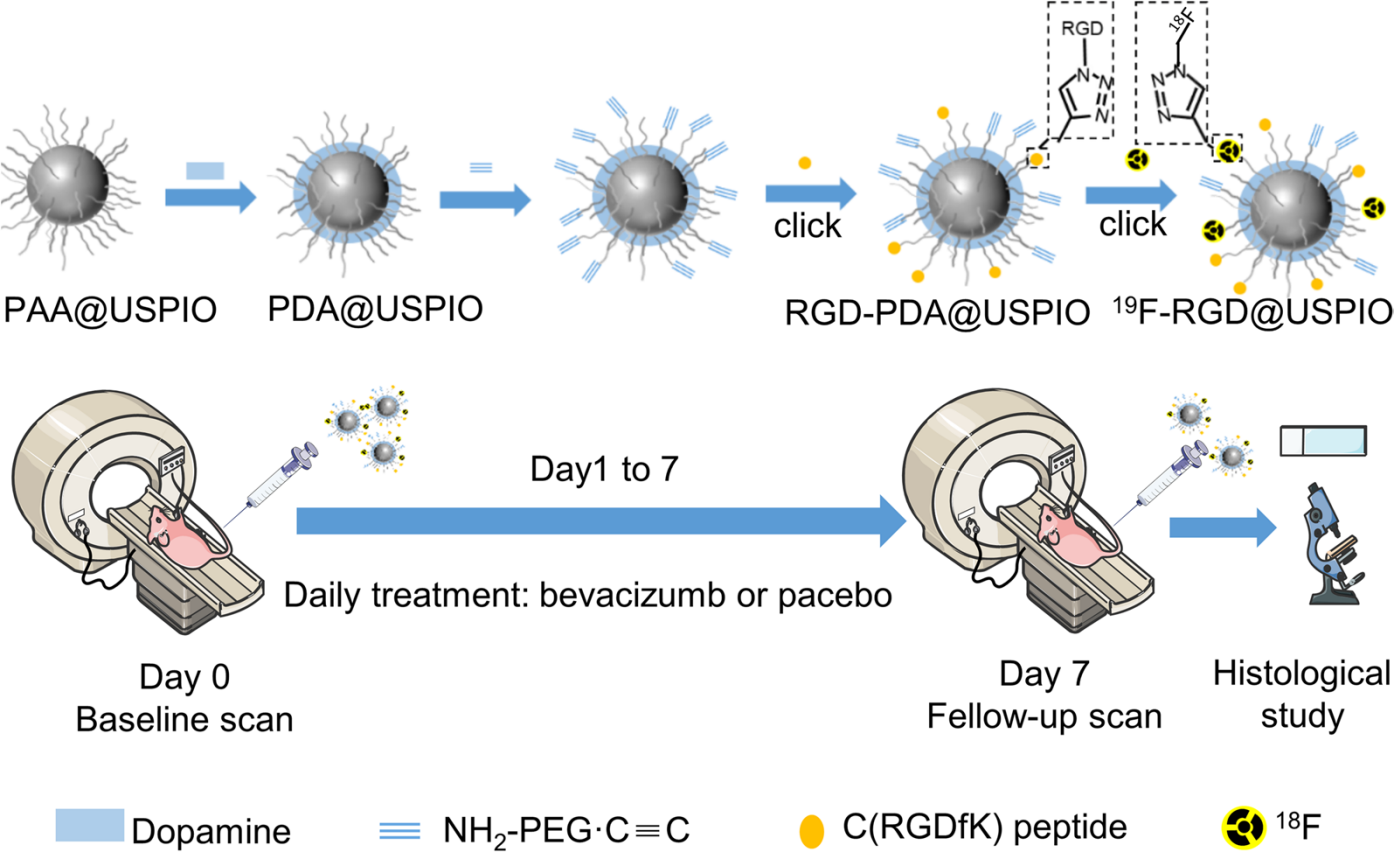
Synthesis and in-depth investigation route of ^18^F-RGD@USPIO

^18^F-RGD@USPIO was synthesized by conjugating RGD peptides and ^18^F to the USPIO (Fig. 1). Iron (III) acetylacetonate (Fe(acac)_3_ ≥ 97%) was purchased from Sigma-Aldrich Chemical Co. Ltd. (St Louis, MO, USA). The PAA-coated USPIO (PAA@USPIO) was synthesized using the polyol method according to previous report [24]. Briefly, poly (acrylic acid) (PAA, 50 wt%, solution in water, MW 1000, Acros, 8 mmol) and anhydrous ferric chloride (FeCl_3_, 4 mmol) were dissolved in 30 mL of DEG (diethylene glycol) and heated in a microwave under vigorous stirring. When the temperature reached 220 °C, sodium hydroxide solution (100 mg/mL, 2 mL) which dissolved in DEG was rapidly injected. After heating for another 10 min in a microwave, the mixture was cooled to the ambient temperature. The as-prepared nanoparticles were precipitated with a mixture of ethanol and ethyl acetate (1: 3, v/v), retrieved by ultrafiltration (NMWL = 300 000, 10,000 rpm) three times and then re-dispersed in water.

Then, dopamine coating was added to the nanoparticle: a total of 300 μL PAA@USPIO solution (3.7 mg iron concentration) and dopamine (6 mg) were dispersed into a mechanically stirred solution of Tris (hydroxymethyl) aminomethane (pH 8.5). PDA can self-polymerize at alkaline pHs on various substrates despite whether the surfaces are hydrophilic or hydrophobic [25]. During polymerization, PDA will spontaneously form a conformal and continuous coating layer atop nearly any material present in the reaction media, and synthetic polymers via the strong binding affinity of catechol functional groups.

After incubating for 12 h, a hetero-biofunctional linker was then added into the mixture solution, and stirred gently for 12 h at room temperature. The linker we used was alkynyl-poly(ethylene glycol)-amine (NH2-PEG-alkyne, MW 3400, Creative PEGWorks, Chapel Hill, NC, 10 mg), which has an alkynyl group at one end and an amino group at the other end. The amine groups could react with the quinone in PDA via the Michael addition reaction, forming an irreversible neighboring covalent bond and thereby anchoring the NH_2_-PEG-alkyne moiety on the surface of PDA@USPIO.

The RGD peptide, c(RGDfK)-N_3_, was synthesized by Chinese Peptides Co., Ltd. (Hangzhou, People’s Republic of China) (Additional file 1: Figure S1). Cu(I)-Catalyzed click chemistry was used for the RGD conjugation. Briefly, a total of 0.0005 mM cyclic RGD peptides c(RGDfK)-N_3_ (abbreviated RGD) in 100–200 μL deionized H_2_O was coupled with alkynes from the mixture solution with the corresponding “click partners”, which contained CuSO_4_ solution (40 mM) and sodium ascorbate solution (120 mM), and then stirred for 30 min at 50 °C. The final nanoparticle, RGD-PDA@USPIO, was retrieved by ultrafiltration (Amicon Ultra-0.5, MWCO 100,000) and washed three times.

The ^18^F ion was obtained by irradiation of liquid ([^18^O]H_2_O]) and adsorbed on a QMA cartridge (Sep-Pak Accell Plus), then eluted with a 1 mL solution that contained kryptofix222 complexes—potassium (14.4 mg) and potassium carbonate (3 mg). For labeling ^18^F by click chemistry, the ^18^F-labeling precursor—2-azidoethyl-4-toluenesulfonate—was first synthesized. Next, ^18^F-labeled 2-[^18^F]fluoroethyl azide ([^18^F]FEA) was prepared through nucleophilic [^18^F] fluorination [26]. Click reaction of 1 mL RGD-PDA@USPIO and [^18^F]FEA was performed in the presence of CuSO_4_/sodium ascorbate at 95 °C for 30 min. Then, the mixture was heated at 50 °C for 30 min. The final product, ^18^F-RGD@USPIO, was retrieved and separated from the unreacted [^18^F]FEA and other impurities by ultrafiltration using size exclusion filters with a 100 kDa molecular weight cutoff until no activity was collected in the filtrate (< 1 µCi). A centrifugal concentrator was then used to concentrate the samples to the desired volume. Figure 1 provides an overview of synthesis and the investigation route.

In addition, RGD peptide-conjugated ^19^F labeled USPIO (^19^F-RGD@USPIO) and nontargeted ^19^F-PDA@USPIO were also prepared as a “cold” probe using ^19^F-FEA as a precursor under the same conditions for ^18^F labeling.

The hydrodynamic size and zeta potentials were measured using ^19^F-RGD@USPIO and ^19^F-PDA@USPIO by a Zetasizer Instruments (NanoZS, Malvern Inc), when particles were dispersed in phosphate buffer saline (PBS, pH 7.4). The size and morphology were characterized by transmission electron microscopy (TEM, JEOL 2010) at an accelerating voltage of 200 kV. To verify polymers surface coating, FTIR analysis was performed using a Nicolet 6700 FTIR spectrometer (Thermo Fisher, USA). The longitudinal and transverse relaxation times of ^19^F-RGD@USPIO of the iron concentration at 0.2 mM, 0.4 mM, 0.6 mM, 0.8 mM, and 1 mM were measured at 1.41 T (60 MHz) on a Bruker mq60 nuclear magnetic resonance analyzer. The T1 and T2 relaxivities (r1, r2) were obtained from the slope of the linear fit of the R1 (1/T1) and R2 (1/T2) relaxation rates versus Fe concentration of the ^19^F-RGD@USPIO. The labeling efficiency of ^18^F-RGD@USPIO was calculated by dividing the radioactivity retained at the origin to the total radioactivity added.

The stability of the probes in different medium was evaluated by monitoring their hydrodynamic size changes during 24 h using DLS.

### Flow cytometry

Human umbilical vein endothelial cells (HUVECs) were obtained from the Chinese Academy of Sciences Committee Type Culture Collection cell bank, cultured in DMEM (Gibco, Paisley, UK) medium. MDA-MB-231 (ATCC, Manassas, VA, USA), the human triple-negative breast cancer cell line, was maintained in DMEM culture medium. The culture media were supplemented with 10% fetal bovine serum (Sigma-Aldrich, AU), and the cells were maintained at 37 °C in a 5% CO_2_ incubator. MDA-MB-231 cells and HUVEC were detached by trypsinization and stained on ice with PE mouse anti-human CD61 (BD Pharmingen™, San Jose, CA, USA) or volume-equivalent PBS for 30 min, respectively. After extensive washing, the fluorescence of 10,000 cells was analyzed by an FACS Calibur flow cytometer (Becton–Dickinson, Rutherford, NJ, USA).

### Prussian blue staining

HUVEC and MDA-MB-231 were diluted to 5000 cells/well in 6-well culture plates with DMEM. After 24 h, cells were washed by PBS (pH 7.4) twice, and incubated with 0.3 mM, 1.0 mM, or 3.0 mM (24, 80, 240 μg Fe/mL) ^19^F-RGD@USPIO for 3 h at 37 °C. Afterwards, the adherent cells were washed with PBS and fixed by 4% paraformaldehyde for 15 min. Later, the fixed cells were incubated with a solution of 2% potassium ferrocyanide and hydrochloric acid (V:V = 1:1) for 10 min, and counterstained with 0.1% nuclear fast red for 5 min.

### In vitro cytotoxicity

The toxicity of ^19^F-RGD@USPIO was tested on MDA-MB-231 line by using CCK-8 assay. The cells were incubated in 96-well culture plates at the density of 5000 cells per well in culture medium. After 24 h, ^19^F-RGD@USPIO at the concentration of 0.25, 0.5, 0.75, 1.0, 1.5, and 3.0 mM (20, 40, 60, 80, 120, 240 μg Fe/mL) were introduced to the medium and incubated for 6 h, 12 h or 24 h, 36 h, and 48 h at 37 °C; each concentration was used under identical conditions in 5 wells. Then, an amount of 10 μL CCK-8 (Beyotime, shanghai, CN) was added to each well, and the cells were incubated for 2 h at 37 °C. The absorbance at 450 nm was used to calculate the cell survival rate.

### Animal tumor model

The female Balb/c nude mice (4–6 weeks old, 18–22 g body weight) were purchased from Shanghai Experimental Animal Center and were maintained in a specific pathogen-free environment. All the experimental protocols were approved by the Ethics Committee of Xinhua Hospital Affiliated to Shanghai Jiao Tong University School of Medicine. All methods were conducted in compliance with the relevant guidelines and regulations of the National Institutes of Health for the Care and Use Committee of Laboratory Animals. A total of 42 female BALB/c nude mice were subcutaneously injected by 0.2 mL serum-free media with a total of 1 × 10^7^ cells of MDA-MB-231 into the right hind flank.

### Bio-distribution and targeting kinetic

Six mice were used for isotope-based assessment of in vivo tracer distribution. Tumor bearing mice were injected with 30 μCi (200 ~ 300 μmol Fe/kg) ^18^F-RGD@USPIO (n = 3) or ^18^F-PDA@USPIO (n = 3) via tail vein. Then the mice were killed and dissected after 2 h. The heart, liver, spleen, lung, kidney, stomach, intestine, brain, bone, muscle, and tumor were weighed in plastic test tubes. The radioactivity was obtained in a well-type scintillation detector along with 3 × 0.5 mL aliquots of the diluted standard representing 100% of the injected dose, and present as the percentage of radioactivity in injected dose per gram of tissue (% ID/g).

For the longer term targeting kinetic study, 10 mice without any treatment were imaged with MRI before and at 0.5, 1, 2, 4, 6, and 24 h after intravenous injection with ^19^F-RGD@USPIO (n = 5) or ^19^F-PDA@USPIO (n = 5) at the same dose of 200 μmol [Fe]/kg (in a total volume of 100 μL). All the MR imaging was performed by using a 3.0 T MR system (Ingenia, Philips, Medical System, Best, Netherlands) with an eight-channel receiver coil of 5.0-cm inner diameter (Chen guang Medical Technologies Co., Shanghai, China). Imaging was performed under isoflurane anesthesia (1.5% isoflurane in oxygen at 2 L/min). Transverse T2-weighted (T2W) fast spin echo and T2 mapping sequence were implemented. The parameters for T2W sequence were as follows: TR = 6000 ms, TE = 68 ms, FOV = 50 × 50 mm^2^, matrix = 144 × 136, NSA = 2, 8–12 slices, slice thickness = 1 mm, and slice interval = 0 mm. T2 mapping was performed for the calculation of T2 values: TR = 2000 ms, TE = 13 ms–78 ms (6 echoes, 13 ms interval between two echoes), FOV = 50 × 50 mm^2^, matrix = 120 × 98, NSA = 1, 8–12 slices, slice thickness = 1 mm, slice interval = 0 mm. For each mouse, the region of interest (ROI) was manually drawn on the center slice of the tumor after identification of the solid part of the tumor on conventional T2WI by two professional radiologists (H.H.L and J.N.L, with 3 and 6 years of experience in MRI analysis, respectively) who were blinded to the experimental design. Later, T2 values of the ROI were automatically calculated by the T2 Map on the dedicated post-processing workstation.

Four mice were used for histological assessment of distribution of ^18^F-RGD@USPIO (n = 2) or ^18^F-PDA@USPIO (n = 2), and the control group (n = 2) were injected with a placebo solution. Tumor bearing mice were killed after 6 h post-injection, and the heart, liver, spleen, lung, kidney, intestine, and tumor were embedded in the paraformaldehyde solution immediately after removal. For tumor, consecutive sections were used for triple histological assessment: using rabbit anti-mouse CD31 (ab28364, 1:50, Abcam, Cambridge, UK) for the demonstration of endothelial cells, rat anti-F4/80 (ab16911, 1:300, Abcam) for the staining of macrophages, and Perls’ Prussian blue for the demonstration of iron (Fe^3+^). Prussian blue staining of heart, liver, spleen, lung, kidney, and intestine tissues were also performed, and the procedure was the same as that described in “Histological study” section.

### Anti-angiogenic treatment and treatment evaluation

A total of 20 mice were used to evaluate the effect of bevacizumab. Tumor size was assessed by caliper measurements every other day, and tumor volume was calculated using the following formula: V = 1/2 × length × width^2^. The xenografts were allowed to grow to a diameter of 0.6–1 cm. Before baseline imaging (day 0), 20 mice were randomly allocated to bevacizumab therapy (n = 10) or the placebo (control) group (n = 10). Mice in the therapy group were treated with bevacizumab (Avastin; Basel, Switzerland; 5 mg/kg body weight) for 7 consecutive days, whereas mice in the control group was treated with a placebo solution (volume-equivalent of 0.9% sodium chloride) at the same time. All mice in the control and treated group received MRI and PET/CT studies before (baseline scan) and 7 days after treatment of bevacizumab or placebo (follow-up scan) by using ^18^F-RGD@USPIO as contrast agent. The MRI protocol is the same as described above for *Targeting kinetic Studies*, and MR images were acquired pre-contrastly and 6 h after injecting intravenously with 30 μCi (200–300 μmol Fe/kg) ^18^F-RGD@USPIO according to the time-activity curve of T2 relaxation time. The change of the T2 rates (∆T2) were obtained before and after contrast agent injection (∆T2 = T2_pre-contrast_ − T2_post-contrast_). The difference of ΔT2 values (∆∆T2) between baseline (day 0) and follow-up (day 7) scan (∆∆T2 = ∆T2_follow-up_ − ∆T2_baseline_) were calculated to quantify the change of ^18^F-RGD@USPIO binding under therapy.

Small-animal PET/CT imaging was performed by using an Inveon system (Siemens Preclinical Solutions, Knoxville, Tennessee, USA). PET scans were acquired 2 h after the injection of ^18^F-RGD@USPIO, followed by 10 min micro-CT scans for anatomic information. The images were reconstructed by a three-dimensional ordered subset expectation maximum (3D OSEM) algorithm in the Siemens Inveon Research Workplace 3.0 (IRW 3.0). CT Volumes-of-Interest (VOI) were manually drawn over the tumor and diaphragm to obtain the maximum standardized uptake value (VOI_maxtumor_) and mean standardized uptake value of muscle (VOI_meanmuscle_). The target-to-background ratio (TBR, Volume-of-Interest [VOI_maxtumor_/VOI_meanmuscle_] was determined as a semi-quantitative measure of tumor radiotracer accumulation. ∆TBR (∆TBR = TBR_follow-up_ − TBR_baseline_) was used to show the difference of TBR between the baseline and follow-up scans.

After the follow-up scan (day 7), all mice were killed, the tumors were explanted for immunohistochemistry and immunofluorescence studies. Figure 1 provides an overview of the experimental design.

### Histological study

Tumors were embedded in the paraformaldehyde solution immediately after removal. To quantify microvascular density (CD31) and tumor cell proliferation (Ki-67), paraffin-embedded tissue sections were dewaxed and rehydrated using standard protocols. Slices were incubated with the primary rabbit anti-mouse CD31 antibody (ab28364, 1:50, Abcam) or rabbit anti-human Ki67 antibody (ab16667, 1:100; Abcam) separately after blocking with 3% BSA for 30 min, and then tissue sections were treated with HRP-conjugated goat anti-rabbit antibody (A0208, 1:100, Beyotime, shanghai, CN) and expression of CD31/Ki-67 was visualized using an DAB-chromogen (Beyotime).

To detect the expression of CD31/α_v_β_3_-integrin fluorescent double staining, frozen tumor tissue sections (5 μm) were thawed at 37 °C for 10 min, followed by slide-fixing with acetone for 10 min and air-drying for 30 min at room temperature. After blocking with 10% goat serum for 1 h, slices were simultaneously incubated with rabbit anti-mouse CD61 (integrin β3) antibody (NBP1-76544, 1:200; Novus Biologicals, Littleton, USA) and rat anti-mouse CD31 antibody (ab7388, 1:300, Abcam), and then visualized with Alexa Fluor@555-conjugated goat anti-rabbit (A27039, 1:300, Invitrogen, Carlsbad, CA, USA) and Alexa Fluor@488-conjugated goat anti-rat secondary antibody (A-11006, 1:300, Invitrogen). DAPI (1:1000, Beyotime) was used for nuclear staining. Fluorescence images were acquired with an epifluorescence microscope (Olympus, X81).

For Prussian blue staining, paraffin-embedded tissue sections were incubated with potassium ferrocyanide solution and hydrochloric acid (V:V = 1:1), and counterstained with 0.1% nuclear fast red for 5 min as described in vitro [27]. The pathologist who performed the immunohistochemistry analysis was blinded to the experimental groups. Microvessel density was determined on a 200× field within the area of most intense tumor neovascularization. The positive area was quantitated in five random high-power fields at 200× magnification using Image J software (NIH, Bethesda).

### Statistical analysis

The statistical analysis was performed using SPSS 23 (IBM Corp, Armonk, NY). Student’s t-test or Mann–Whitney U-test were applied for intra- and inter-group comparisons of the quantitative parameters. Linear correlations between non-normally distributed variables were assessed by Spearman’s test. The level of significance was set at *P*-value < 0.05.

## Results

### Characterization of ^19^F-RGD@USPIO and ^19^F-PDA@USPIO

**Fig. 2:**
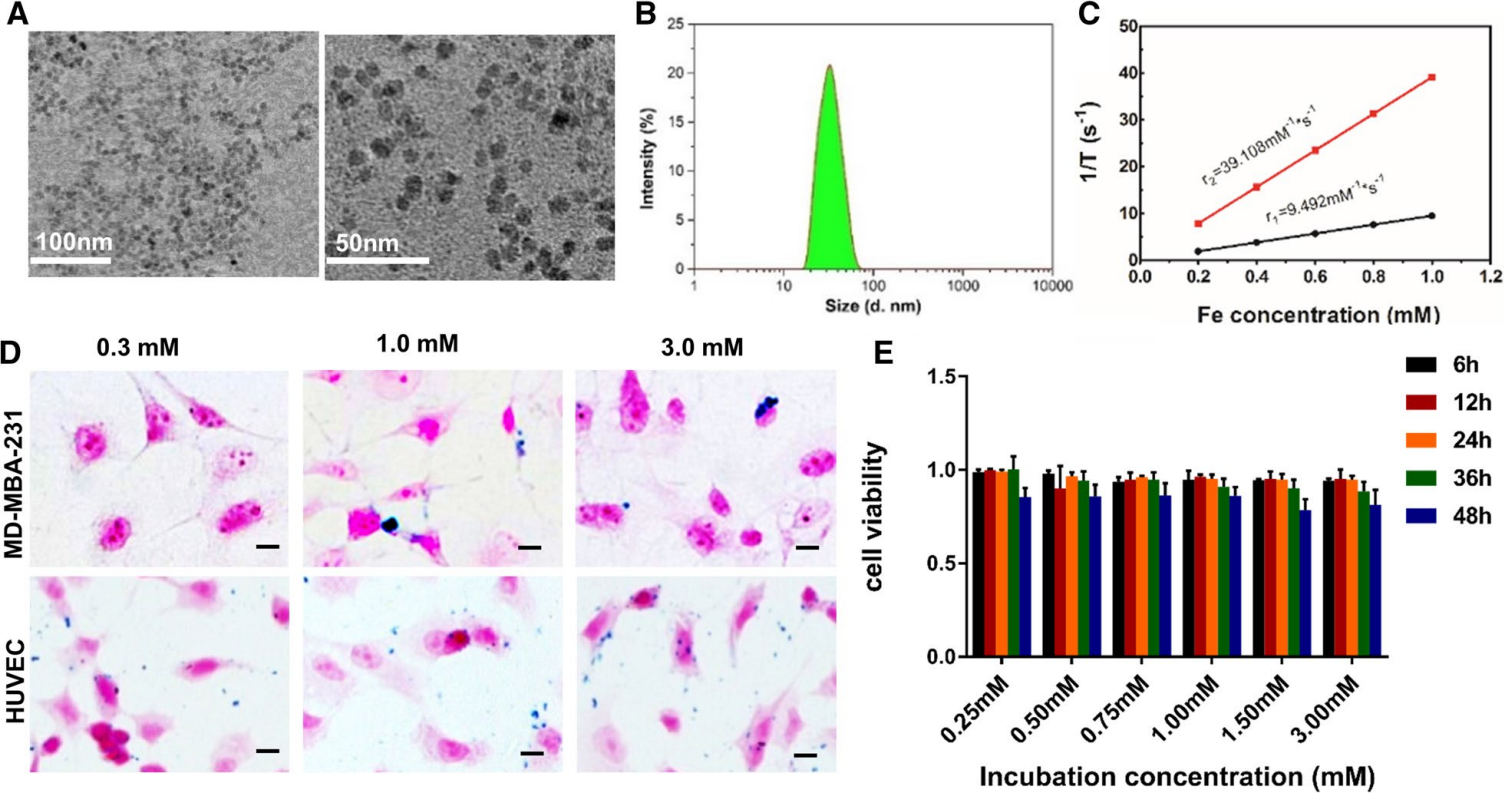
Characterization of ^19^F-RGD@USPIO probes. **A** Representative TEM images of ^19^F-RGD@USPIO nanoparticles. **B** Size distribution of ^19^F-RGD@USPIO measured by dynamic light scattering system. **C** Relaxation properties of ^19^F-RGD@USPIO. Prussian blue staining of HUVEC and MDA-MB-231 cells incubated with ^19^F-RGD@USPIO (**D**). Magnification ×200. Cytotoxicity characteristics of different concentrations of ^19^F-RGD@USPIO after 6 h, 12 h, 24 h, 36 h and 48 h incubation with MDA-MB-231 cells, error bars denote standard errors (**E**)

The core size of the ^19^F-RGD@USPIO determined by TEM was 6.5 ± 0.5 nm (Fig. 2A). PDA@USPIOs was uniform in size, and was stable in physiological conditions (Additional file 2: Figure S2, Additional file 3: Figure S3). DLS analysis showed that the mean hydrodynamic diameter of ^19^F-RGD@USPIO was 40.44 nm (Fig. 2B), and the mean hydrodynamic diameter of ^19^F-PDA@USPIO was 39.77 nm (Additional file 2: Figure S2). After PEG coating, the zeta potential decreased from − 11.51 to − 20.73 mV. The longitudinal (r1) and transversal (r2) relaxivities of ^18^F-RGD@USPIO were 9.49 and 39.11 s^−1^ mM^−1^, respectively (Fig. 2C). FTIR was shown in Additional file 4: Figure S4, the peak around 3200–3300 cm^−1^ was attributed to the absorbed water, and that at 620–625 cm^−1^ was characteristic of Alkyen groups. The radiochemical yield was 22.61%. The particles were stable in physiological conditions (Additional file 3: Figure S3).

### Cytotoxicity of ^19^F-RGD@USPIO

CCK-8 assay showed that at different concentrations (0.25, 0.5, 0.75, 1.0, 1.5, 3.0 mM), the cytotoxicity of the probes was not significant, with cell viability still greater than 80%. These results showed ^19^F-RGD@USPIO exhibited low cytotoxicity (Fig. 2E).

### Integrin a_v_β_3_ expression and binding assays in vitro

The FACS analysis demonstrated that the positive rate of MDA-MB-231 cells stained with PE mouse anti-human CD61 was only 6.5%, as compared to 44.5% of HUVECs (Additional file 5: Figure S5A, B). HUVEC exhibited significant ^19^F-RGD@USPIO binding, whereas MDA-MB-231 cells demonstrated only marginal ^19^F-RGD@USPIO binding (Fig. 2D). ^19^F-RGD@USPIO binding to HUVEC increased with higher ^19^F-RGD@USPIO concentrations (3.0 mM > 1.0 mM > 0.3 mM).

### Long term targeting kinetic study

**Fig. 3:**
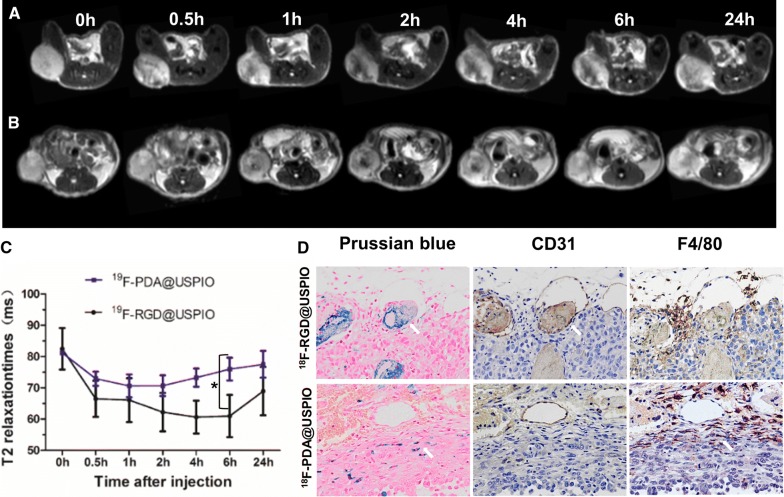
Specificity evaluation of the probe in vivo and ex vivo. T2-weighted MR imaging of tumor before and 0.5, 1, 2, 4, 6, and 24 h after intravenous injection of ^19^F-RGD@USPIO (**A**) or nontargeted ^19^F-PDA@USPIO (**B**). **C** Time-activity curves of T2 relaxation time of tumors before and after probe injection. Error bars denote standard errors, Mann–Whitney U-test were applied, *P < 0.05. **D** Prussian blue iron staining, CD31 vascular staining, and F4/80 macrophage staining were performed in consecutive sections: (antigen-positive area are brown and USPIO-positive area are blue: see arrows). While ^18^F-RGD@USPIO and ^18^F-PDA@USPIO both accumulate within tumors, the ^18^F-PDA@USPIO do so mainly through association with macrophages rather than microvasculature. Magnification: × 200

To investigate the a_v_β_3_-integrin targeting ability and optimize the time point for therapeutic effect evaluation, in vivo T2-weighted fast spin-echo and T2 mapping sequence was performed with mice bearing MDA-MB-231 tumors for long term (24 h) targeting kinetic study (Fig. 3A, B). After injection with ^19^F-RGD@USPIO via the tail vein, MRI signal intensity decrease was found in the tumor. Accordingly, quantitative analysis of T2 relaxation time after injection of ^19^F-RGD@USPIO or nontargeted control ^19^F-PDA@USPIO is shown in Fig. 3C. The time activity curves of ^19^F-RGD@USPIO and ^19^F-PDA@USPIO accumulation were different. There was no significant difference at pre-contrast (82.49 ± 6.65 ms vs. 81.18 ± 0.72 ms, P = 0.685). The accumulation of ^19^F-RGD@USPIO was faster than ^19^F-PDA@USPIO within the first 30 min post-injection of nanoprobes (66.50 ± 5.76 ms vs. 72.93 ± 2.28 ms, P = 0.049). However, there was no significant difference between ^19^F-RGD@USPIO and non-targeted ^19^F-PDA@USPIO at the 1-h point (66.10 ± 7.02 ms vs. 70.64 ± 3.65 ms, P = 0.235). After 2 h, a continuous accumulation of the ^19^F-RGD@USPIO was observed over 6 h, while ^19^F-PDA@USPIO was clear from the tumor tissue. The T2 relaxation time was significantly different at 6 h (60.98 ± 6.74 ms vs. 75.99 ± 3.64 ms, P = 0.002).

Additionally, Prussian blue staining of tumor tissues showed large amount of ^19^F-RGD@USPIO distributed in the interior of the blood vessels (CD31-positive area) around the periphery of tumor, while subtle ^19^F-PDA@USPIO were mainly distributed in the interstitial space around the tumor and probably colocalizated with macrophages (Fig. 3D).

### In vivo distribution

**Fig. 4:**
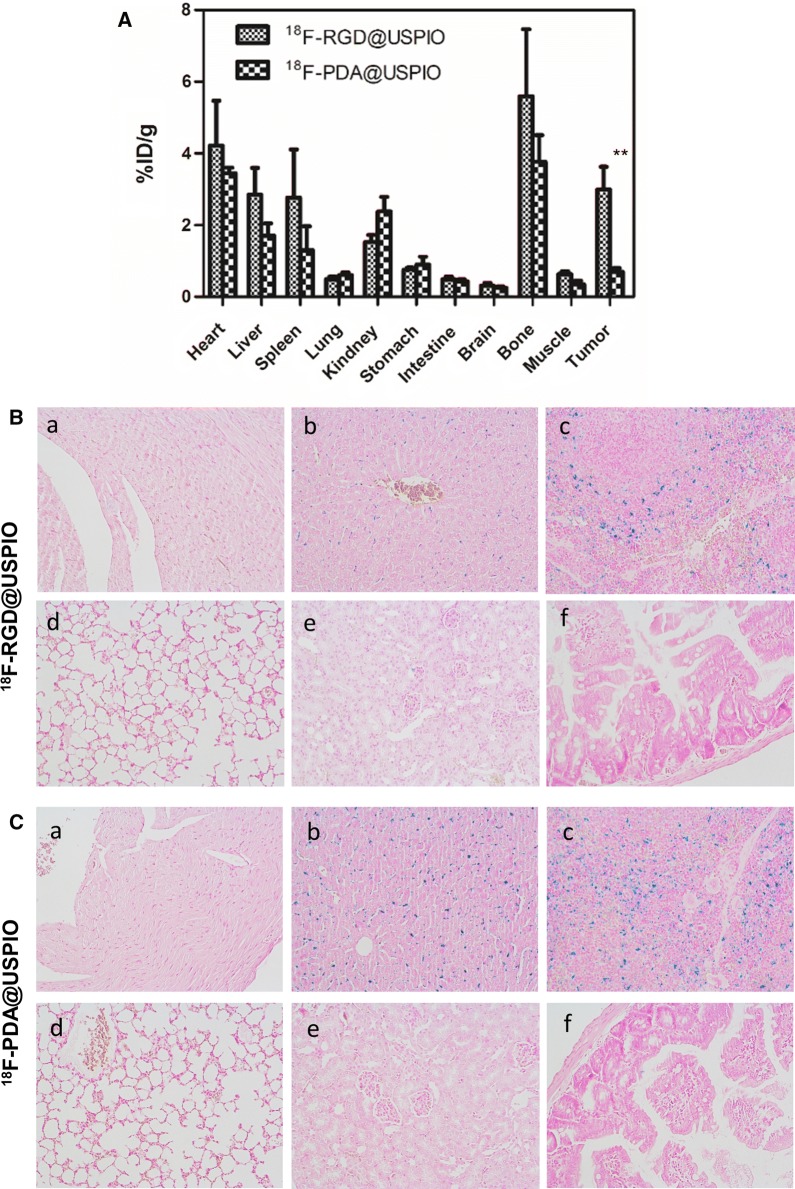
Bio-distribution of ^18^F-RGD@USPIO and non-targeted control. **A** Bio-distribution of ^18^F-PDA@USPIO and ^18^F-RGD@USPIO 2 h after injection. Error bars denote standard errors, Mann–Whitney U-test were applied, ** P < 0.01. Prussian blue staining of heart (a), liver (b), spleen (c), lung (d), kidney (e), intestine (f) sections after injection of ^18^F-RGD@USPIO (**B**) and ^18^F-PDA@USPIO (**C**). Magnification: × 200

Tissue distribution of ^18^F labeled nanoprobes in MDA-MB-231 bearing mice bearing are shown in Fig. 4. Tumor uptake of ^18^F-RGD@USPIO was significantly higher than non-targeted ^18^F-PDA@USPIO (2.71 ± 0.63 vs. 0.98 ± 0.19%ID/g, P < 0.01) according to isotope-based bio-distribution study 2 h post-injection (Fig. 4A). Among the organs evaluated, bone showed the highest uptake of ^18^F-RGD@USPIO (5.36 ± 1.18%ID/g) (Fig.4A).

The Fe stained in liver (Fig. 4B-b) and spleen (Fig. 4B-c) were obvious by Prussian blue staining. The low amount of iron found in kidney (Fig. 4B-e) by Prussian blue staining stand for absent ^18^F-RGD-USPIO accumulation in kidney, like heart (Fig. 4B-a), lung (Fig. 4B-d) and intestine (Fig. 4B-f). The accumulation of ^18^F-PDA-USPIO in organ (Fig. 4C) is the same as ^18^F-RGD-USPIO. For control group (Additional file 6: Figure S6), the Fe staining in kidney, heart, lung intestine, and tumor is absent. The Prussian blue staining in liver (Additional file 6: Figure S6-b) and spleen (Additional file 6: Figure S6-c) revealed a significant lowering in control group than the ^18^F-RGD-USPIO and ^18^F-PDA-USPIO, and we can conclude that the Fe stained in liver and spleen is not endogenous.

### Effects of bevacizumab on tumor volume

There was no significant difference in baseline tumor volumes between the therapy and control group (volume_therapy-baseline_ = 163.54 ± 90.02 mm^3^, volume_control-baseline_ = 141.50 ± 50.42 mm^3^; P = 0.508). As displayed in Additional file 7: Figure S7, although less time-dependent increase in tumor volume was observed in the treated group than the control group, there was no significant intergroup difference in tumor volume change after therapy onset (Δvolume_therapy_ = 127.31 ± 63.77 mm^3^, Δvolume_control_ = 200.28 ± 122.60 mm^3^; P = 0.112).

### Monitoring the anti-angiogenic effect of bevacizumab by MRI and PET/CT imaging with ^18^F-RGD@USPIO

**Fig. 5:**
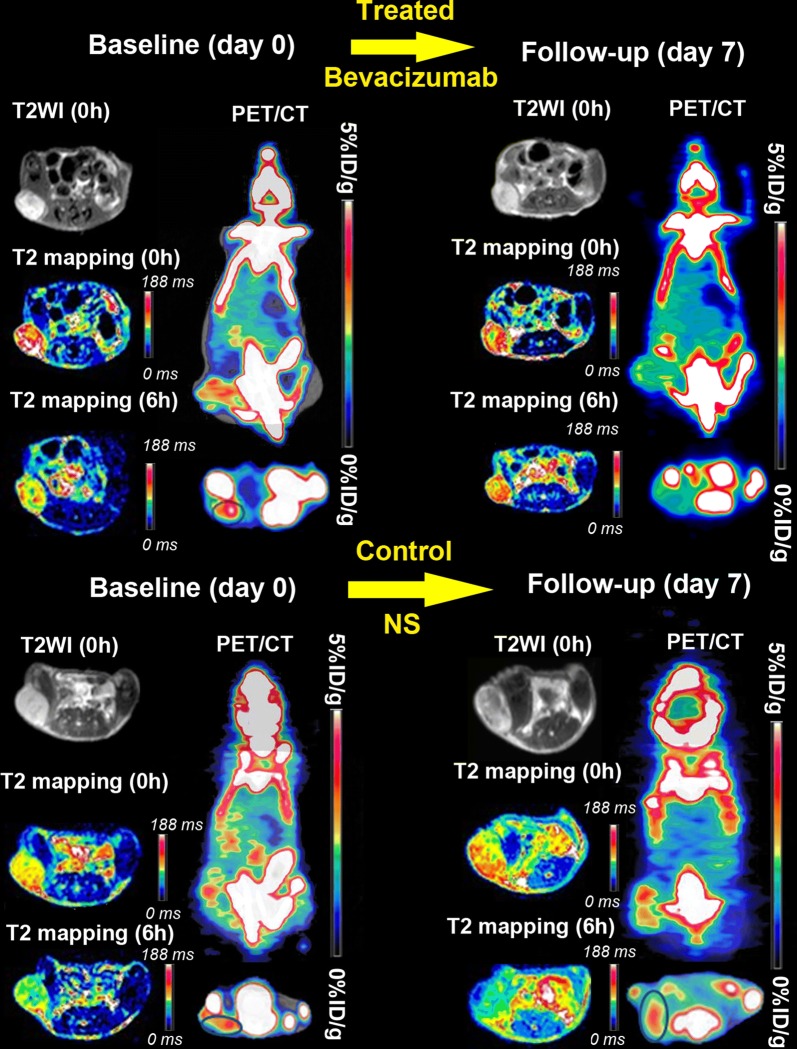
Representative T2WI, T2 mapping and PET/CT images of mice in the control and treatment groups after injection of ^18^F-RGD@USPIO

**Fig. 6:**
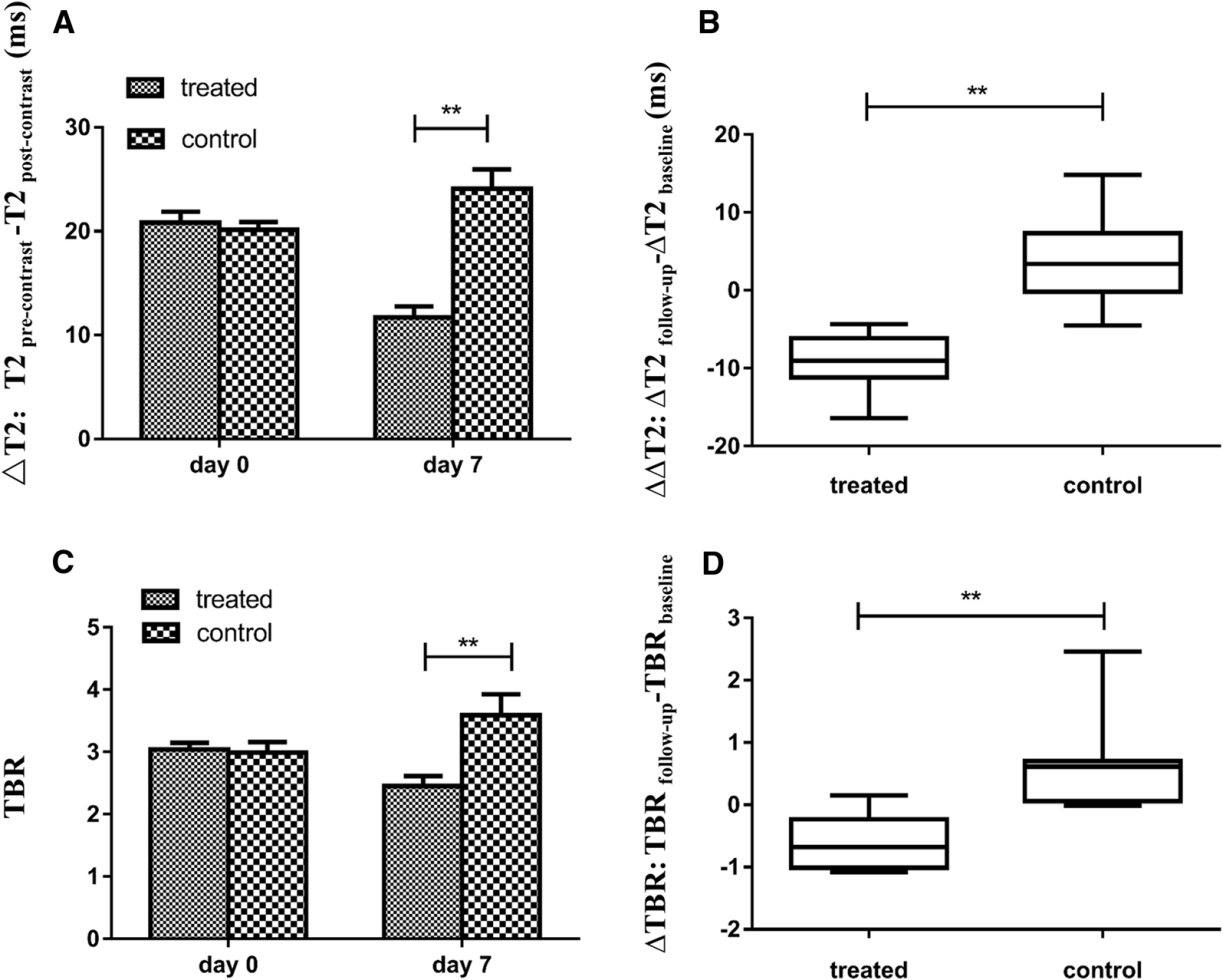
Quantitative analysis of the decrease of T2 relaxation time and TBR values of tumors between control and treatment groups after injection of ^18^F-RGD@USPIO. **A** ΔT2 at baseline (Day 0, 6 h post-contrast) and follow-up (Day 7). **B** The change of ΔT2 values at Day 7 compared to baseline measurement (ΔΔT2). **C** TBR at baseline (Day 0, 2 h post-injection) and follow-up (Day 7). **D** The change of TBR between baseline and follow-up. Error bars denote standard errors, Mann–Whitney U-test were applied **P < 0.05

The MRI and PET/CT imaging at baseline (day 0) and follow-up (day 7) are shown in Fig. 5. There was no significant intergroup difference on day 0 both in MRI (Fig. 6A, ∆T2, 20.84 ± 3.30 ms vs. 20.12 ± 2.39 ms, P = 0.586) and PET/CT (Fig. 6C, TBR, 3.04 ± 0.33 vs. 2.99 ± 0.54, P = 0.799). We observed a significant decline in follow-up ∆T2 values compared with the baseline values in the therapy group (Fig. 6A, ∆T2, 20.84 ± 3.30 ms vs. 11.72 ± 3.28 ms, P < 0.01), but increase in the control group (Fig. 6A, ∆T2, 20.12 ± 2.39 ms vs. 24.09 ± 5.93 ms, P = 0.056). The therapy group showed significantly lower ∆∆T2 than the control group (Fig. 6B, ∆∆T2, − 9.12 ± 3.55 ms vs. 3.96 ± 5.69 ms, P < 0.01). TBR showed an obvious decrease in the therapy group (Fig. 6C, TBR, 3.04 ± 0.33 vs. 2.45 ± 0.51, P = 0.002), whereas significantly increased in control group (TBR, 2.99 ± 0.54 vs. 3.59 ± 1.07, P = 0.027); ∆TBR was significantly different following treatment (Fig. 6D, mean ΔTBR: − 0.59 ± 0.43 vs. 0.60 ± 0.72; P < 0.01).

### Ex vivo tumor tissue analyses for assessing response to bevacizumab treatment

**Fig. 7:**
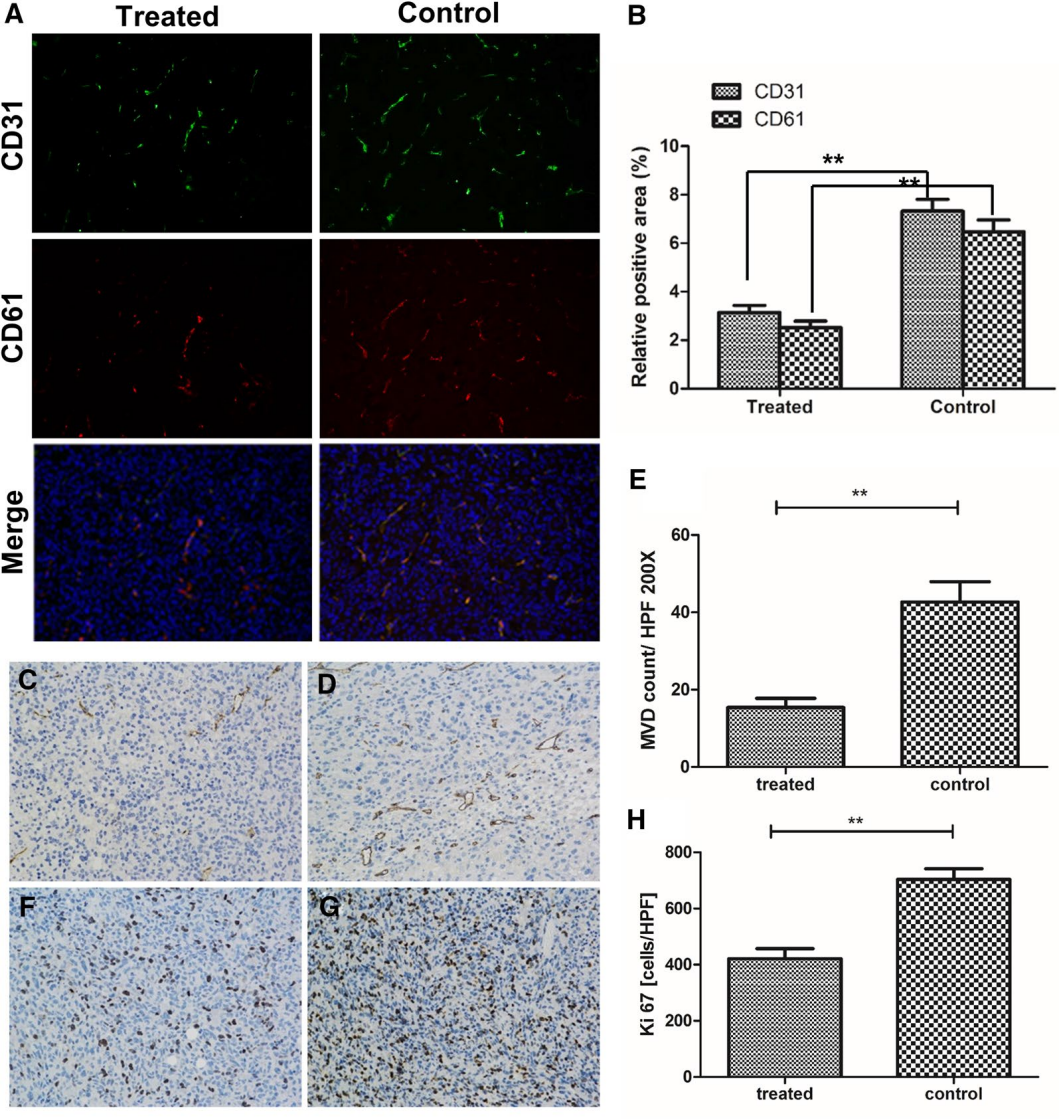
Histological analyses for assessing response to bevacizumab treatment. **A** Immunofluorescence staining of tumor vasculature and integrin β_3_ with antibodies against CD31 (green) and CD61 (integrin β_3_, red) in the treated and control groups at the end of the study. **B** Quantitative analysis of integrin β_3_ and CD31-positive area fraction of tumors between the control and treatment groups. **P < 0.05. Magnification: ×200. Microvascular density (CD31) from the therapy group (**C**) and control group (**D**). Tumor cell proliferation (Ki-67) from the therapy group (**F**) and control group (**G**). Quantitative MVD (**E**) and Ki67 (**H**) assessment of therapy and control group. Error bars denote standard errors, Mann–Whitney U-test were applied, **P < 0.05. Magnification: ×200

Immunohistochemistry revealed a significant lowering of microvascular density (Fig. 7C–E, CD31, 15.38 ± 7.62 vs. 42.68 ± 16.64; P < 0.01) and proliferation (Fig. 7F–H, Ki-67, 421.08 ± 113.77 vs. 703.50 ± 120.03; P < 0.01) in the therapy group than the control group. A similar trend was found for the integrin β3 expression (Fig. 7A, B, CD61 positive area, 2.52.0.84% vs. 6.471.56%, P < 0.01).

## Discussion

Although many interesting multimodal imaging probes have been suggested, their spatiotemporal characteristics have been investigated using each imaging device separately, making the exact correlation and comparison studies impossible. With the development of preclinical and clinical simultaneous PET/MRI systems, developing dual-modality PET/MRI probes has been a much debated and researched topic since their emergence in the last decade. ^68^Ga and ^64^Cu were the most commonly used PET tracers among the reported PET/MRI nanoprobes [28]. However, It is known that ^18^F is the most popular radioisotope for routine clinical use with distinct advantages: first, ^18^F has the lowest maximum positron energies among the positron-emitting PET tracers, which means the total absorbed dose of the subject and the loss in spatial resolution are minimum; second, ^18^F was produced from inexpensive precursors [28]; furthermore, ^18^F has optimal half-lives (t_1/2_ = 1.83 h) when compared with ^64^Cu (t_1/2_ = 12.70 h) and ^68^Ga (t_1/2_ = 1.13 h). Unfortunately, there were few reports of ^18^F-labeled nanoparticles, as adding ^18^F to functionally complex materials is chemically challenging [29].

In our study, we developed a labeling platform that can achieve rapid labeling of ^18^F by Click Chemistry, and successfully assembled a nanoprobe with well-controlled size and high relaxivity. The radio-isotope incorporation, ligand exchange, and purification were accomplished within 1 h, which is appropriate for 1.83 h half-life of ^18^F. Therefore, ^18^F-RGD@USPIO has a high potential for clinical applications. However, the high uptake of radio-isotope in bone in our study may indicate that the purification from the nonreacted [^18^F] FEA is incomplete, and the improvement of purification remains to be an ongoing research.

Thus far, most studies of dual-modality PET/MRI probes have limited themselves at the level of lesion detection [28]. As the hybrid imaging strategy is superior to single modality in providing information, research not only for detection but also for performance in-depth investigation on real world applications should be warranted, such as cancer staging and monitoring the effects of treatment [23]. Hence, our study aimed to demonstrate the feasibility and effectiveness of ^18^F-RGD@USPIO to assess early anti-angiogenic therapeutic effects in experimental breast cancer. With high spatial resolution, the MRI could provide the detailed anatomic and probe distribution information after anti-angiogenic treatment. However, in terms of sensitivity, the use of PET expands, by one or two orders of magnitude, the concentration limit that can be used compared to MRI. Furthermore, when using USPIO for “negative” contrast in MRI, the identification of the negative signal coming from the nanoparticles is not always straightforward. The incorporation of PET signal helps for much easier detection of the lesion, especially for real-world process characteristics of many focuses, assisting the resolution of whole-body MRI and detecting the change of anti-angiogenic therapy sensitively. After accurately obtaining the in vivo distribution of probes, we can get a range of functional information not only by conventional T2WI and T2 mapping, but also by using other functional MR sequence after probe injection, such as SWI [20], CEST [30], and T1rho [31].

The breast cancer cell line MDA-MB-231 has a low α_v_ß_3_-integrin expression (Additional file 1: Figure S1), thereby facilitating the imaging of α_v_ß_3_-integrin expressing as a biomarker of tumor microvasculature in MDA-MB-231 tumor model [16]. Prussian blue staining that was used to visualize iron oxide deposits, demonstrated the co-localization of ^18^F-RGD@USPIO with the vessel endothelial cells in vitro and ex vivo. Afterwards, ^18^F-RGD@USPIO enabled visualization of tumor angiogenesis.

Before clinical application, the in vivo kinetic of ^18^F-RGD@USPIO need to be extensively studied. The predominant mechanism of nanoparticle accumulation in tumors is enhanced permeability and retention effect (EPR) [32], but this mechanism is detrimental in obtaining biomarker-specific information. Thus, choosing the best time point is important in monitoring the dynamic expression of a biomarker. As integrin α_v_β_3_ expression at endothelial cells is directly accessible in the blood, targeting accumulation of RGD-functionalized nanoparticles is faster than the passive accumulation owing to the EPR effect. Some studies [33, 34] suggest early-stage imaging will be advantageous to obtain target-specific information. However, nanoprobes in blood will present significant background signals at early times. Our study compared the pre- and post-contrast images of ^19^F-RGD@USPIO at various time points on MRI over non-targeted control for seeking the best one. We chose “cold” probe for the evaluation length (24 h) is not compatible with the half-life of ^18^F. Our study shows the accumulation of ^19^F-RGD@USPIO was faster than ^19^F-PDA@USPIO within the first 30 min post-injection of nanoprobes. However, there was no significant difference between ^19^F-RGD@USPIO and non-targeted ^19^F-PDA@USPIO at the 1-h point, which may be because of passive accumulation. After 2 h, a continuous accumulation of the ^19^F-RGD@USPIO was observed for over 6 h while ^19^F-PDA@USPIO was clear from the tumor tissue, which may be because RGD-conjugated nanoparticles have higher affinity for target molecules and could remain longer within the target tissues. In the histological analysis, which were performed to uncover more subtle differences in overall tumor targeting than could be done by imaging, showed that the targeted probe accumulated at the microvasculature after 6-h post injection, while the control probe was found to extravasate into the interstitial space. The accumulation of ^18^F-PDA@USPIO was less epithelium-specific than that for ^18^F-RGD@USPIO and was dependent on a combination of EPR and phagocytosis which was even more importantly by tumor associated macrophages. Taken together, to simultaneously obtain target-specific information and present significant tumor-to-background signals, we chose 6-h post-injection as the time point to perform an MRI scan.

Taking into consideration with in vivo degradation of ^18^F, the PET/CT image was acquired 2 h post-injection, for enabling much easier detection of the lesion sensitivity. Because the degradation of ^18^F is on their way, and the radiotracer uptake is normalized to muscle (diaphragm), we chose TBR as the semi-quantitative measure for therapeutic effect evaluation. TBR is less sensitive to inter- and intra-individual variations in mouse weight, radiotracer blood clearance, and radiotracer dose when compared with the standardized uptake value.

In the previous studies, ex vivo immunohistochemistry revealed a significantly reduced of density of tumor microvasculature (MVD) and α_v_β_3_-integrin in MDA-MB-231 xenograft-bearing mice after VEGF inhibition [16]. Binding of RGD-based nanoprobes is modulated by expression and activation status of α_v_β_3_-integrin [35]. Accordingly, we found ^18^F-RGD@USPIO binding was significantly decreased in the bevacizumab-treated group, while it was increased in the control group, consistent with immunohistochemistry findings. In the present investigation, several differences can be acknowledged by monitoring the early antiangiogenic effects with single modality probe. First, larger nanoprobes facilitate the targeting of intravascular, that is, endothelial structures, as they bear lower probability of nonspecific extravasation and α_v_β_3_-expressed tumor cells binding. The optimal size of ^18^F-RGD@USPIO overcomes limitations of RGD-based PET probes. Second, the simultaneous detection of ^18^F-RGD@USPIO at the same site on both PET/CT and MRI can enhance the sensitivity and specificity of the probe’s accumulation when compared to using MRI alone: MR image signal does not originate from the nanoparticles, rather from the interaction of hydrogen protons and nanoparticles, and this interaction may be changed by a multitude of effects within the biological tissues [36]. The contrast provided by ^18^F could ensure the existence of the probe. In light of this, ^18^F-RGD@USPIO is more precise in identifying in advance how bevacizumab responds before there is no change in tumor size.

Our study had several limitations: First, in vivo imaging of α_v_β_3_-integrin expression was only assessed before and after treatment. However, anti-angiogenesis is a complex biological process: bevacizumab could induce transient normalization of the tumor-associated blood vessels, thereby improving delivery and efficacy of chemotherapy; vessel co-option or lining of tumor channels by endothelial cells may cause resistance to bevacizumab [37]. RGD-based probes have been used to investigate some differences for angiogenesis phenotyping of tumors [34]. It is hence imaginable that the dual-modality molecular probe could not only image in basal conditions and after therapy but also monitor complex dynamic changes during anti-angiogenic therapy. Additional imaging time points over the course of treatment may allow for a more accurate depiction of α_v_β_3_-integrin under therapy. Second, EPR effect cannot be completely excluded as detailed understanding of ^18^F-RGD@USPIO targeting kinetics is lacking. It would be useful to synthesize a dual-modality probe with a concentration range that would be an acceptable compromise for simultaneous PET/MRI. Hence the radiolabeling method and surface functionalization need further investigation to ensure the stability of ^18^F-RGD@USPIO.

## Conclusion

In our study, we demonstrated that both PET/CT and MRI with ^18^F-labeled, RGD-coupled, PAA-coated USPIO allowed in vivo assessment of anti-angiogenesis therapeutic effects by targeting α_v_β_3_-integrin in a human breast xenograft model. Furthermore, this PET/MRI dual modality contrast agent could likely predict which patients could benefit from bevacizumab in breast cancer, and add complementary molecular imaging of therapeutic effects to morphology-based and functional tumor response assessments.

## Supplementary information

**Additional file 1: Figure S1.** Scheme of c(RGDfK)-N_3._

**Additional file 2: Figure S2.** Hydrodiameter of ^19^F-PDA@USPIO.

**Additional file 3: Figure S3.** The stability of the probes in different medium.

**Additional file 4: Figure S4.** FTIR of PDA@USPIO and PDA@USPIO-Alkyne.

**Additional file 5: Figure S5.** Quantification of CD61 (integrin ß3) expression on HUVEC and MDA-MB-231 cells using flow cytometry.

**Additional file 6: Figure S6.** Prussian blue staining of heart (a), liver (b), spleen (c), lung (d), kidney (e), intestine (f) and tumor (g) after injection of placebo solution. Magnification: ×200.

**Additional file 7: Figure S7.** Tumor volume over the course of the experiments. Comparison of tumor volumes in the control and bevacizumab-treated group in MD-MBA-231 xenograft model. Tumor volume was determined by caliper measurements.

## Data Availability

All data generated or analyzed during this study are included in this published article and its additional information files.

## References

[1] DeSantis CE, Ma J, Goding SA, Newman LA, Jemal A (2017). Breast cancer statistics, 2017, racial disparity in mortality by state. CA Cancer J Clin.

[2] Ribatti D, Nico B, Ruggieri S, Tamma R, Simone G, Mangia A (2016). Angiogenesis and antiangiogenesis in triple-negative breast cancer. Transl Oncol.

[3] Zambonin V, De Toma A, Carbognin L (2017). Clinical results of randomized trials and ‘real-world’ data exploring the impact of bevacizumab for breast cancer: opportunities for clinical practice and perspectives for research. Expert Opin Biol Ther.

[4] Wong PP, Bodrug N, Hodivala-Dilke KM (2016). Exploring novel methods for modulating tumor blood vessels in cancer treatment. Curr Biol.

[5] Shin S, Noh Y (2018). Increased risk of adverse drug events secondary to bevacizumab treatment in patients with advanced or metastatic breast cancer: a meta-analysis of randomized controlled trials. Ther Clin Risk Manag.

[6] Tirumani SH, Fairchild A, Krajewski KM (2015). Anti-VEGF molecular targeted therapies in common solid malignancies: comprehensive update for radiologists. Radiographics.

[7] Plow EF, Meller J, Byzova TV (2014). Integrin function in vascular biology: a view from 2013. Curr Opin Hematol.

[8] Chakravarty R, Chakraborty S, Dash A (2015). Molecular imaging of breast cancer: role of RGD peptides. Mini Rev Med Chem.

[9] Rylova SN, Barnucz E, Fani M (2014). Does imaging alphavbeta3 integrin expression with PET detect changes in angiogenesis during bevacizumab therapy?. J Nucl Med.

[10] Kazmierczak PM, Todica A, Gildehaus FJ (2016). 68 Ga-TRAP-(RGD)3 hybrid imaging for the in vivo monitoring of alphavss3-integrin expression as biomarker of anti-angiogenic therapy effects in experimental breast cancer. PLoS ONE.

[11] Minamimoto R, Karam A, Jamali M (2016). Pilot prospective evaluation of (18)F-FPPRGD2 PET/CT in patients with cervical and ovarian cancer. Eur J Nucl Med Mol Imaging.

[12] Iagaru A, Mosci C, Mittra E (2016). Glioblastoma multiforme recurrence: an exploratory study of (18)F FPPRGD2 PET/CT. Radiology.

[13] Terry SY, Abiraj K, Lok J (2014). Can 111In-RGD2 monitor response to therapy in head and neck tumor xenografts?. J Nucl Med.

[14] Becker S, Bohn P, Bouyeure-Petit AC (2015). Bevacizumab enhances efficiency of radiotherapy in a lung adenocarcinoma rodent model: role of alphavbeta3 imaging in determining optimal window. Nucl Med Biol.

[15] Chen WT, Shih TT, Chen RC, Tu SY, Hsieh WY, Yang PC (2012). Integrin alphavbeta3-targeted dynamic contrast-enhanced magnetic resonance imaging using a gadolinium-loaded polyethylene gycol-dendrimer-cyclic RGD conjugate to evaluate tumor angiogenesis and to assess early antiangiogenic treatment response in a mouse xenograft tumor model. Mol Imaging.

[16] Kazmierczak PM, Schneider M, Habereder T (2016). alphavss3-integrin-targeted magnetic resonance imaging for the assessment of early antiangiogenic therapy effects in orthotopic breast cancer xenografts. Invest Radiol.

[17] Sirsi SR, Flexman ML, Vlachos F (2012). Contrast ultrasound imaging for identification of early responder tumor models to anti-angiogenic therapy. Ultrasound Med Biol.

[18] Zhou Y, Shao G, Liu S (2012). Monitoring breast tumor lung metastasis by U-SPECT-II/CT with an integrin alpha(v)beta(3)-targeted radiotracer(99m)Tc-3P-RGD(2). Theranostics.

[19] Shao G, Gu W, Guo M (2017). Clinical study of (99m)Tc-3P-RGD2 peptide imaging in osteolytic bone metastasis. Oncotarget.

[20] Yang S, Lin J, Lu F, Han Z, Fu C, Gu H (2017). Use of ultrasmall superparamagnetic iron oxide enhanced susceptibility weighted imaging and mean vessel density imaging to monitor antiangiogenic effects of sorafenib on experimental hepatocellular carcinoma. Contrast Media Mol Imaging.

[21] Yang SH, Lin J, Lu F (2016). Contrast-enhanced susceptibility weighted imaging with ultrasmall superparamagnetic iron oxide improves the detection of tumor vascularity in a hepatocellular carcinoma nude mouse model. J Magn Reson Imaging.

[22] Kumar A, Zhang S, Hao G (2015). Molecular platform for design and synthesis of targeted dual-modality imaging probes. Bioconjug Chem.

[23] Garcia J, Tang T, Louie AY (2015). Nanoparticle-based multimodal PET/MRI probes. Nanomedicine.

[24] Yang C, Mi X, Su H (2019). GE11-PDA-Pt@USPIOs nano-formulation for relief of tumor hypoxia and MRI/PAI-guided tumor radio-chemotherapy. Biomater Sci.

[25] Liu X, Cao J, Li H (2013). Mussel-inspired polydopamine: a biocompatible and ultrastable coating for nanoparticles in vivo. ACS Nano.

[26] Li J, Shi L, Jia L (2012). Radiolabeling of RGD peptide and preliminary biological evaluation in mice bearing U87MG tumors. Bioorg Med Chem.

[27] Cui Y, Zhang C, Luo R (2016). Noninvasive monitoring of early antiangiogenic therapy response in human nasopharyngeal carcinoma xenograft model using MRI with RGD-conjugated ultrasmall superparamagnetic iron oxide nanoparticles. Int J Nanomed.

[28] Lahooti A, Sarkar S, Laurent S, Shanehsazzadeh S (2016). Dual nano-sized contrast agents in PET/MRI: a systematic review. Contrast Media Mol Imaging.

[29] Jacobson O, Kiesewetter DO, Chen X (2015). Fluorine-18 radiochemistry, labeling strategies and synthetic routes. Bioconjug Chem.

[30] Chen Z, Yan C, Yan S (2018). Non-invasive monitoring of in vivo hydrogel degradation and cartilage regeneration by multiparametric MR imaging. Theranostics.

[31] Moonen RP, van der Tol P, Hectors SJ, Starmans LW, Nicolay K, Strijkers GJ (2015). Spin-lock MR enhances the detection sensitivity of superparamagnetic iron oxide particles. Magn Reson Med.

[32] Golombek SK, May JN, Theek B (2018). Tumor targeting via EPR: strategies to enhance patient responses. Adv Drug Deliv Rev.

[33] Kessinger CW, Togao O, Khemtong C, Huang G, Takahashi M, Gao J (2011). Investigation of in vivo targeting kinetics of alpha(v)beta(3)-specific superparamagnetic nanoprobes by time-resolved MRI. Theranostics.

[34] Jarzyna PA, Deddens LH, Kann BH (2012). Tumor angiogenesis phenotyping by nanoparticle-facilitated magnetic resonance and near-infrared fluorescence molecular imaging. Neoplasia.

[35] Andriu A, Crockett J, Dall’Angelo S, Piras M, Zanda M, Fleming IN (2018). Binding of alphavbeta3 integrin-specific radiotracers is modulated by both integrin expression level and activation status. Mol Imaging Biol.

[36] Hoffman D, Sun M, Yang L (2014). Intrinsically radiolabelled [(59)Fe]-SPIONs for dual MRI/radionuclide detection. Am J Nucl Med Mol Imaging.

[37] De Palma M, Biziato D, Petrova TV (2017). Microenvironmental regulation of tumour angiogenesis. Nat Rev Cancer.

