# Supplementary figures and images for: 18F-labeled magnetic nanoparticles for monitoring anti-angiogenic therapeutic effects in breast cancer xenografts
Source: J Nanobiotechnology. 2019 Oct 11;17:105. doi: 10.1186/s12951-019-0534-7 (PMC6788012; doi:10.1186/s12951-019-0534-7)

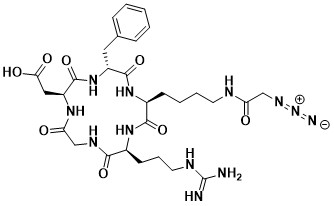

Supplement: Supplementary file 1 — Additional file 1: Figure S1. Scheme of c(RGDfK)-N3. [file 12951_2019_534_MOESM1_ESM.jpg]

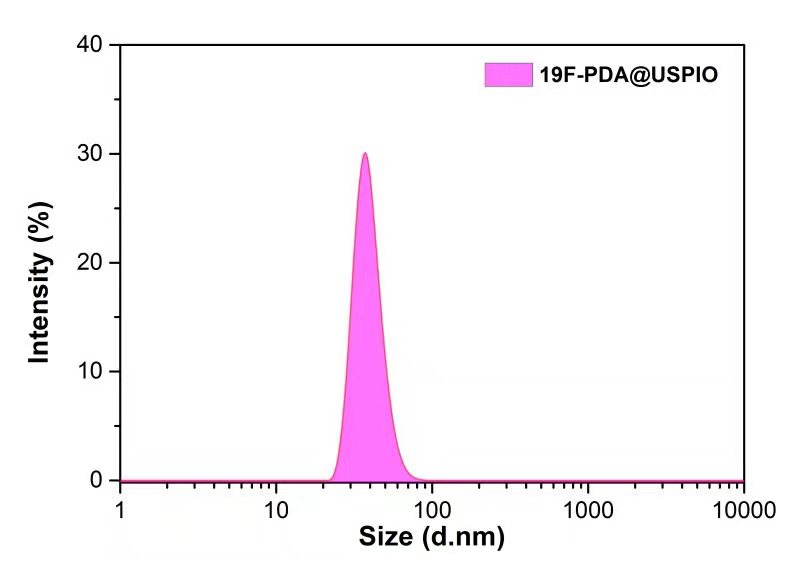

Supplement: Supplementary file 2 — Additional file 2: Figure S2. Hydrodiameter of 19F-PDA@USPIO. [file 12951_2019_534_MOESM2_ESM.jpg]

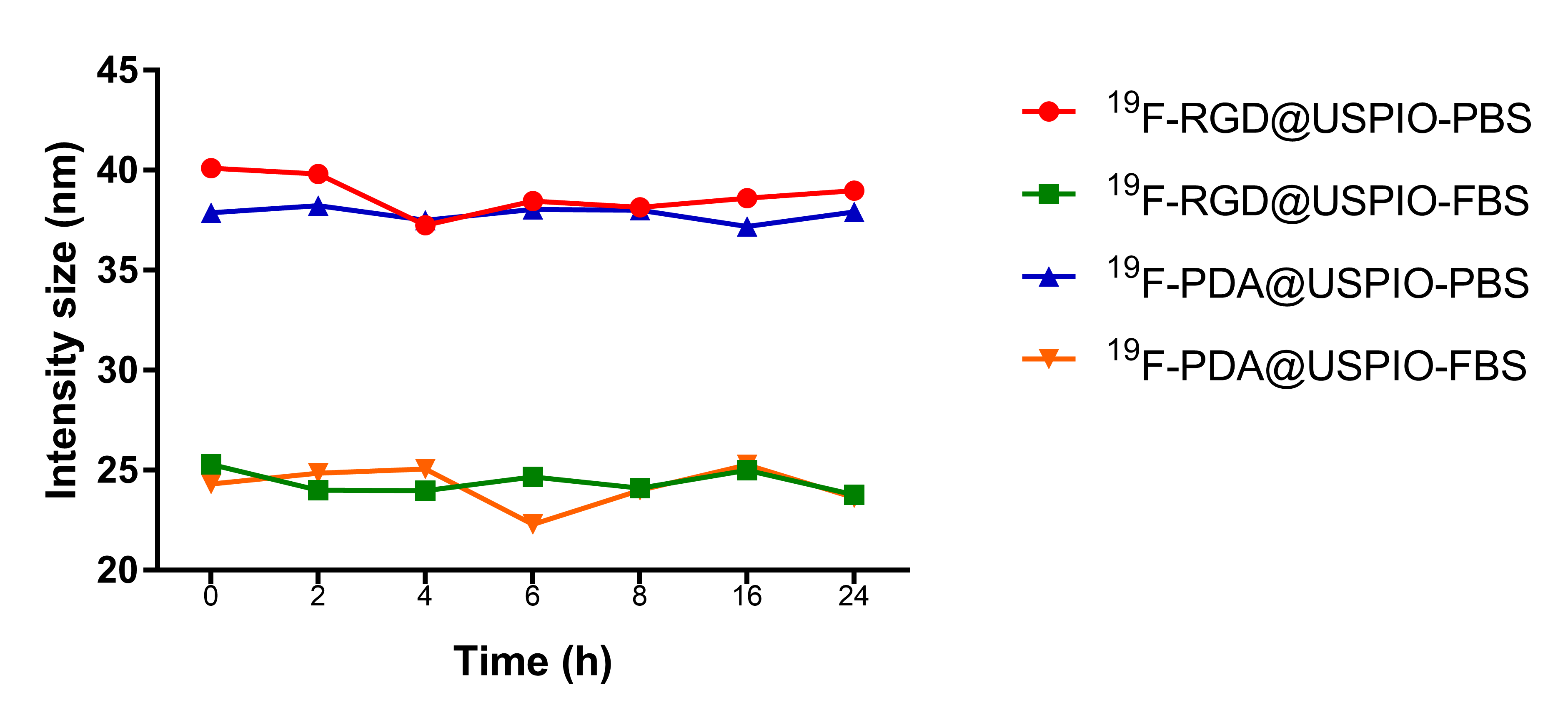

Supplement: Supplementary file 3 — Additional file 3: Figure S3. The stability of the probes in different medium. [file 12951_2019_534_MOESM3_ESM.tif]

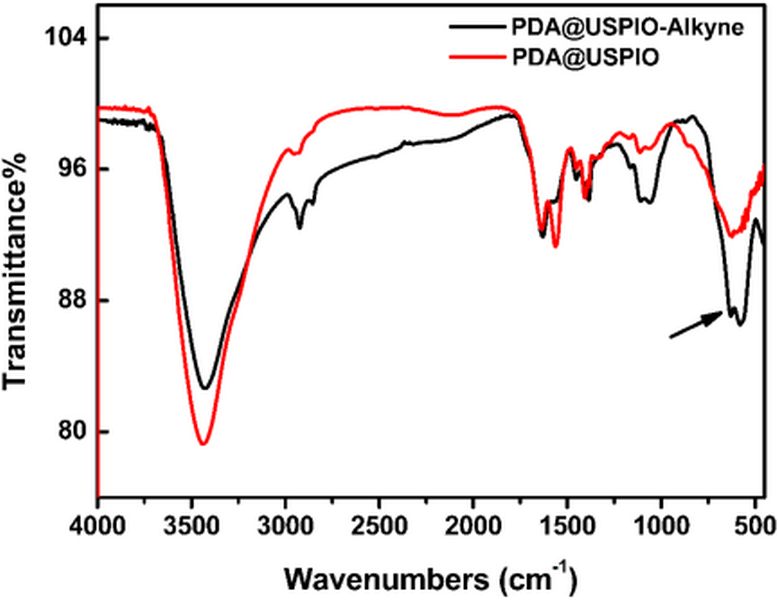

Supplement: Supplementary file 4 — Additional file 4: Figure S4. FTIR of PDA@USPIO and PDA@USPIO-Alkyne. [file 12951_2019_534_MOESM4_ESM.tif]

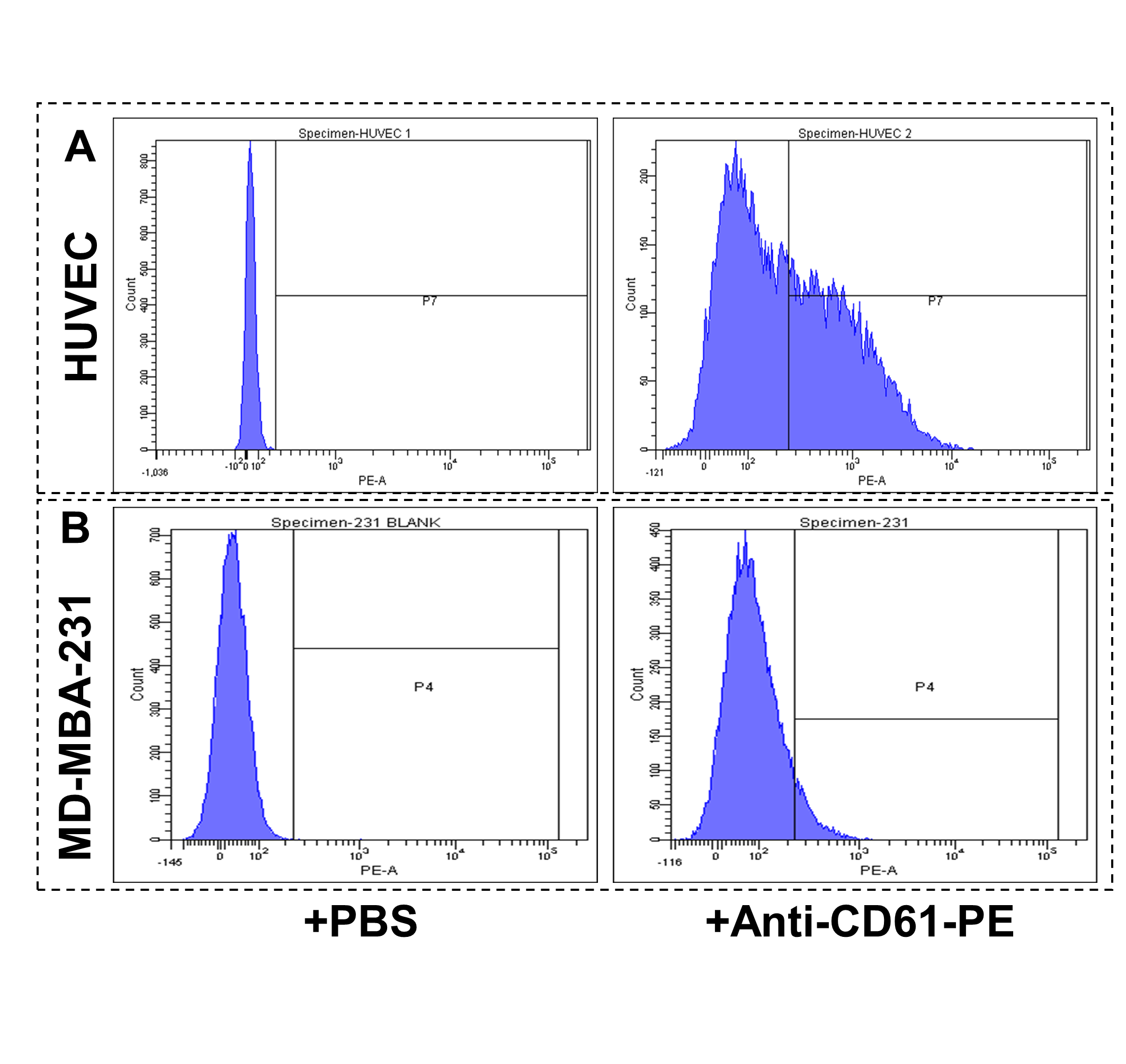

Supplement: Supplementary file 5 — Additional file 5: Figure S5. Quantification of CD61 (integrin ß3) expression on HUVEC and MDA-MB-231 cells using flow cytometry. [file 12951_2019_534_MOESM5_ESM.tif]

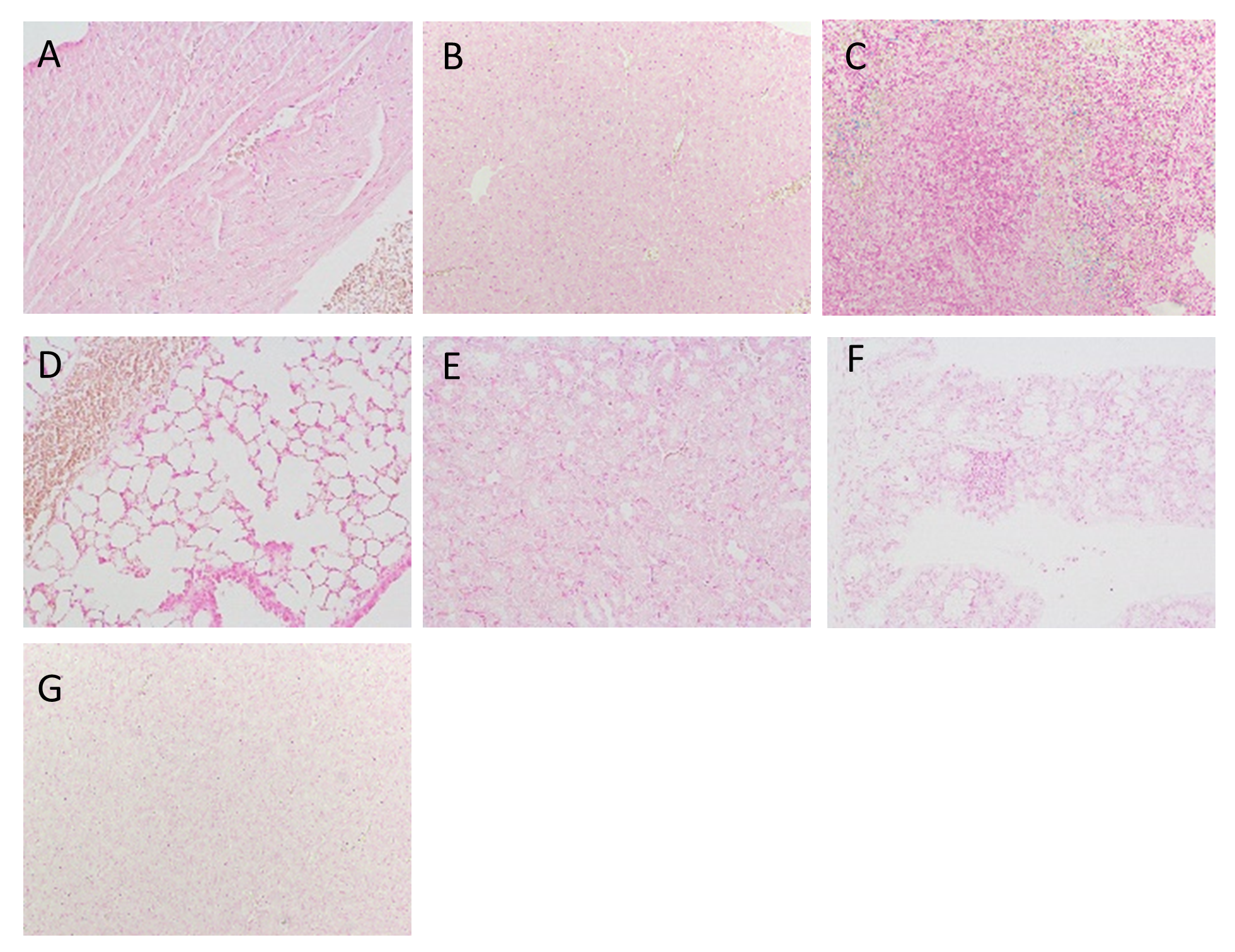

Supplement: Supplementary file 6 — Additional file 6: Figure S6. Prussian blue staining of heart (a), liver (b), spleen (c), lung (d), kidney (e), intestine (f) and tumor (g) after injection of placebo solution. Magnification: ×200. [file 12951_2019_534_MOESM6_ESM.bmp]

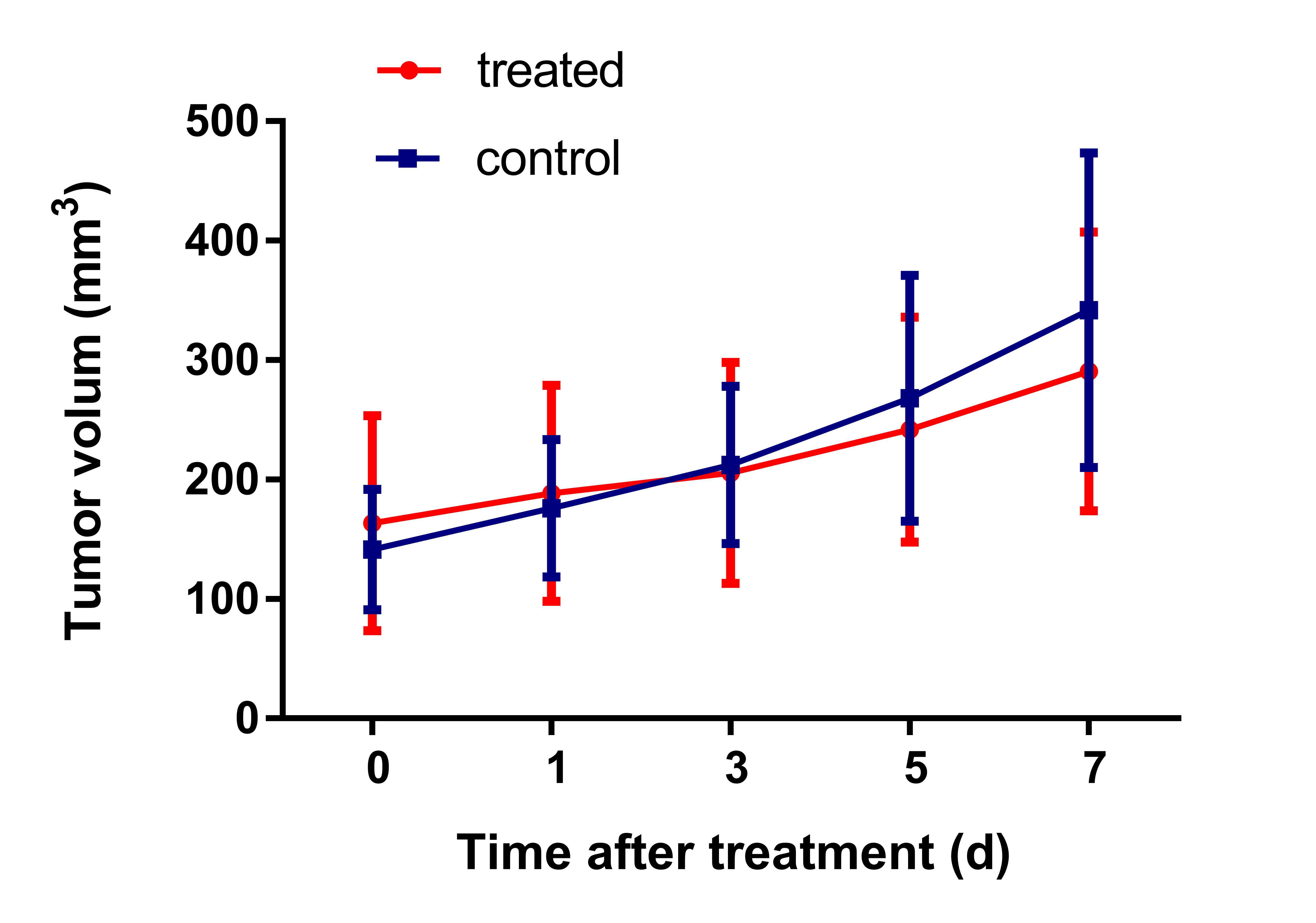

Supplement: Supplementary file 7 — Additional file 7: Figure S7. Tumor volume over the course of the experiments. Comparison of tumor volumes in the control and bevacizumab-treated group in MD-MBA-231 xenograft model. Tumor volume was determined by caliper measurements. [file 12951_2019_534_MOESM7_ESM.tif]
